# Risk factors of surgical site infection post cesarean section

**DOI:** 10.1186/1753-6561-5-S6-P189

**Published:** 2011-06-29

**Authors:** MA Alishaq, JA AlAjmi, B Al-Ali, F Saleh, M El-Sheik, M Malkawi, A George, L Garcia, B Locus

**Affiliations:** 1 Risk Managment .quality, HMC Qatar, Doha, Qatar; 2 Medicine, HMC Qatar, Doha, Qatar

## Introduction / objectives

The risk factors for post cesarean surgical site infection (SSI) have not been identified, although the occurrence of SSI is increasing.

## Objective

To identify the high risk factors of post cesarean section SSI.

## Methods

Retrospective study was conducted at WH. This included a total of 1806 women who underwent to C-section from Jan 2008 to Dec 2009. The risk factor data were collected from I.C surveillance notification form of each patient’s SSI. The risk factors which were studied included: age, SSI bundles, type of C-section elective or emergency, duration of operation (less or more than 57 minutes), SSI index score, receiving anti-biotic as prophylaxis, length of stay pre/post surgery, PROM, comorbidity.

## Results

During 2Yrs study period, 1806 patients underwent CS, of those 82 patients met the case definition for SSI with onset of infection within 30 days after CS. Over the study period, of those 82, 74 (90.2%) were classified as superficial incision, 6 (7.3%) had deep incision SSI, and 2 (2.4%) had organ space infection. Over the study period, 82 infected CS cases, 65 were underwent emergency CS, giving an overall SSI incidence (79.2%).

10 risk factors were studied such as emergency operation, co-morbidity as obesity and diabetes, premature rupture of membranes, prolonged operative time, length of stay pre/post surgery, lack of implementing SSI bundle such as Chlorohexidine skin preparation and hair removal. The emergency CS and obesity s were identified as the high risk factors for acquiring SSI post CS. The other factors were not shown to be significantly high risk factors as timing of antibiotic prophylaxis and duration of operation.

## Conclusion

The finding shows that the 2 high risk factors make difference that lead to different approach concerning wt control, implementing SSI bundle properly and following the IC standards for skin preparation.

## Disclosure of interest

None declared.

